# Large Cohort Data Based Cost-Effective Disease Prevention Design Strategy: Strong Heart Study

**DOI:** 10.4236/wjcd.2018.812058

**Published:** 2018-12-29

**Authors:** Wenyu Wang, Elisa T. Lee, Barbara V. Howard, Richard Devereux, Ying Zhang, Julie A. Stoner

**Affiliations:** 1 Center for American Indian Health Research, Hudson College of Public Health, University of Oklahoma Health Sciences Center, Oklahoma City, OK, USA; 2 MedStar Research Institute, Washington DC, USA; 3 Cornell University Medical Center, New York, NY, USA

**Keywords:** Cost-Effective, Costs-Benefits-Balanced Selecting, Disease Prevention, Prevention Design, Prevention Strategy, Translate Study to Clinical Practice

## Abstract

**Background and Objective:**

A multitude of large cohort studies have collected data on incidence and covariates/risk factors of various chronic diseases. However, approaches for utilization of these large data and translation of the valuable results to inform and guide clinical disease prevention practice are not well developed. In this paper, we proposed, based on large cohort study data, a novel conceptual cost-effective disease prevention design strategy for a target group when it is not affordable to include everyone in the target group for intervention.

**Methods and Results:**

Data from American Indian participants (n = 3516; 2056 women) aged 45 – 74 years in the Strong Heart Study, the diabetes risk prediction model from the study, a utility function, and regression models were used. A conceptual cost-effective disease prevention design strategy based on large cohort data was initiated. The application of the proposed strategy for diabetes prevention was illustrated.

**Discussion:**

The strategy may provide reasonable solutions to address cost-effective prevention design issues. These issues include complex associations of a disease with its significant risk factors, cost-effectively selecting individuals at high risk of developing disease to undergo intervention, individual differences in health conditions, choosing intervention risk factors and setting their appropriate, attainable, gradual and adaptive goal levels for different subgroups, and assessing effectiveness of the prevention program.

**Conclusions:**

The strategy and methods shown in the illustrative example can also be analogously adopted and applied to other diseases preventions. The proposed strategy provides a way to translate and apply epidemiological study results to clinical disease prevention practice.

## 1. Introduction

Prevention of chronic diseases has emerged as an urgent issue due to increasing prevalence of the chronic diseases and their effects on medical care, public health and economic burden. For example, it is estimated that >18 million Americans have diabetes (DM) and are at risk of related vascular complications [1]. Current treatments of DM are only partially successful in preventing its progression and complications. Therefore, early interventions are desirable to reduce DM-related complications and costs of medical care. Several studies/trials have showed that DM may be prevented/delayed either through lifestyle or pharmacological interventions [2] [3] [4]. However, many important issues in designing an effective prevention program have not been considered or discussed sufficiently. These issues include complex associations of a disease with its combined and correlated risk factors, identifying individuals for intervention if the intervention is not affordable for the entire target group, individual differences in health conditions, and selecting risk factors to target with interventions and setting appropriate treatment goal levels. On the other hand, large cohort studies have derived many results and collected datasets for incidence and covariates/risk factors of different diseases. Development of methods for utilization of these valuable results and costly collected data in designing more cost-effective and efficient disease prevention is still ongoing. In this paper, we proposed a conceptual cost-effective disease prevention strategy that might provide reasonable solutions to the aforementioned issues, and demonstrated through simulation how the proposed strategy could be applied to prevent DM in American Indians (AI) based on the available data from the Strong Heart Study (SHS) [5]. The SHS is a population-based cohort study of cardiovascular disease (CVD) and its risk factors for American Indians in southwestern Oklahoma, central Arizona, and North and South Dakota.

## 2. Methods

Let us consider designing a disease prevention program to reduce incident risk of a disease in a given time period, say, four years, for a group/community (called **the target group**) in a population for which it is not affordable to include everyone in the target group for intervention. We will use the following example to show the related issues in the design, and how to use available data from a large cohort study that includes the same or a similar group that is representative of the target group in terms of the factors considered (called **the reference group**) in the prevention design.

### Example:

Consider a DM (defined as having a fasting plasma glucose (FPG) > 126 mg/dl or hemoglobin A1c (HbAlc) ≥ 6.5%) prevention in the target group (aged 40+ years AI with a waist circumference (WAIST) > 102 cm and free of DM).

### Available result:

The following SHS DM risk (probability) prediction model [6] (or the respective DM risk-calculator at https://strongheartstudy.org/Community/Risk-Calculators).

(1) P(an individual will develop DM in four years)=1/(1+exp(−xbeta))

where

(2) $$\begin{array}{l} xbeta=11.3544-0.0292\;\times \text{Age}+0.0167\;\times \;\text{WAIST}+0.2856\;\times \;I\left(\text{elevated blood pressure}\right)\\ \;+\;0.0002\;\times \text{FPG × FPG}-6.4798\;\times \text{HbAlc}+0.6856\;\times \text{HbAlc}\times \text{HbAlc}\\ \;+\;0.0192\;\times \text{Log}\left(\text{UACR}\right)\;\times \;\mathrm{Log}\;\left(\text{UACR}\right)+0.3723\;\times \;I\left(\text{hypertriglyceridemia}\right) \end{array}$$

and in which the “elevated blood pressure” is defined as systolic blood pressure (SBP)/diastolic blood pressure (DBP) ≥ 130/85 mmHg or on hypertension (HTN) medication treatments, UACR denotes urinary albumin/creatinine ratio, hypertriglyceridemia is defined as triglyceride (TG) ≥ 150 mg/dl, and *I*(.) the indicator function (for example, I(hypertriglyceridemia) = 1 if hypertriglyceridemia presented; =0 otherwise).

### Already collected data:

Data from **the reference group** (the SHS baseline (1989–1991) AI participants, aged 45 – 75 years, with WAIST > 102 cm and free of DM).

### 2.1. Identifying Individuals for Intervention if It Is Not Affordable to Include Everyone in the Target Group for Intervention

It would be desirable to include everyone in the target group for intervention. However, this could be expensive and labor-intensive due to the size of the target group (based on the SHS data, about 46% of aged 40+ non-DM AI may have WAIST > 102 cm, which is huge even from a small community). In addition, not everyone in the target group will develop DM (only about 29% of AI in the target group would develop DM in 4 years based on the SHS data). Therefore, ideally, only those persons who are at high risk of developing DM (or an affordable number within the budget limitation) would receive the intervention. To implement this approach we need to solve **Problem 1**. How to identify those at high risk of developing DM in the target group for intervention? Incident DM is usually the result of combined effects of many risk factors such as FPG, HbA1c, WAIST, UACR, and metabolic syndrome traits, and usually most of them are correlated [6] [7] [8] [9] [10]. Thus, using one or two its risk factors to determine who is at high risk of developing DM may not be appropriate. We propose to use the SHS DM risk (probability) prediction model in Equation (1) to assess the risk. This is because the predicted probability represents optimal combined effects of the major and significant DM risk factors. However, a predicted probability shows only the chance that an individual will develop DM based on his/her current measurements of the risk factors. It does not indicate whether the risk is high enough to warrant intervention. Therefore, a cutoff point for the predicted probability is needed, and those with predicted probability higher than or equal to the cutoff point will be classified as “at high risk of developing DM” or “positive”. To determine the cutoff point, one needs to consider also whether the classification is cost-effective since a lower cutoff point means more individuals will be classified as positive and will undergo the designed intervention and the costs would be increased [11] [12] [13]. To find the optimal cutoff probability, we propose to use the data from the reference group and the following Equation (3), which is a utility function [14] [15] that balances the “costs” of including a false-positive (the 2nd term in the right side of Equation (3)) and the “benefits” of including a true-positive in intervention (the 1st term).

(3) U(p,CIDM, Costs, Benefits)=CIDM×SEN(p)×Benefits−(1−CIDM)×(1−SPE(p))×Costs

Or, equivalenty,

(3a) U(p,CIDM, CBR)=CIDM × SEN(p)−(1−CIDM)×(1−SPE(p))×CBR

where CIDM is the estimated cumulative incidence of DM for the target group (=0.2888 estimated based on the data from the reference group); CBR = Costs/Benefits is a given costs-to-benefits ratio; *p* denotes a cutoff probability, say, *p* = 0.1 to 0.9 by 0.0001; SEN(*p*) and SPE(*p*) are the respective sensitivity and specificity for a given *p*(*i.e*., relating to the accuracy of identifying those who will or will not develop incident DM) and can be obtained based on the data from the reference group and the SHS DM risk prediction model.

For a given estimated CIDM, if CBR has been assumed/estimated for the intervention, the utility can be calculated at each *p* between 0.1 and 0.9. The optimal costs-benefits-balanced cutoff probability associated with the given CBR, denoted as p* is defined as the cutoff probability with the highest utility, that is,

(4) $$U\left(p^{*},\text{CIDM, CBR}\right)\;=\text{max}\{U\;\left(p,\text{CIDM, CBR}\right)\;,0\;<p<\;1\}$$

In a special case when CBR equals CIDM/(1-CIDM) (that is the odds of DM), from Equation (3a) and (4), the corresponding p* also maximizes SEN(*p*) + SPE(*p*).

In the case that funds are budgeted to have only a fixed number of individuals in the target group for the intervention, the affordable cutoff probability *p*^†^ can be simply estimated as

(5) $$p^{†}=\text{the}\left\{100\times \left(1-\frac{\text{The fixed number in the target group for intervention}}{\text{Estimated total number of individuals in the target group}}\right)\right\}\text{percentile of “all predicted probabilities from the AIs in the reference group”}$$

After identified participants for intervention based on either the optimal costs-benefits-balanced cutoff probability p* or the affordable cutoff probability *p*^†^, we encountered immediately **Problem 2.** How to choose disease risk factors to address with intervention, and determine their appropriate, attainable and safe goal levels? As we aforementioned, incident DM is usually the result of combined effects of many risk factors. Therefore, a prevention program focused on one or two risk factors may not be sufficient, and thus may decrease efficacy of the program. Furthermore, the usual way to set one uniform goal for a risk factor for all participants in a prevention program may not be appropriate or attainable due to individual differences in risk factors and health conditions, and sometimes may even cause adverse effects and safety problems. Adverse events, medication toxicity, and safety problems are reasons that some clinical trials are discontinued. On the other hand, to reduce risk of a disease for those “at high risk of developing DM” or “positive” individuals in the target group through a prevention program, one intuitive way is to improve the profiles of risk factors of the disease in the “positive” individuals to the profiles in the others who are “not-positive” in the target group. To implement these considerations and the approach, we adopted ways from our previous paper [16] to conduct simultaneous intervention for all of the significant risk factors in the disease prediction model, and use the following methods to derive goal levels for each of the risk factors based on the data from the reference group.

### 2.2. Derive Goal Levels of All Risk Factors in the Disease Risk Prediction Model

To reduce effects of individual differences in risk factors and health conditions on setting goal levels for each of the risk factors, we divide all individuals in the reference group into subgroups based on some of the major risk factors in the prediction model, and derive goal levels for each of the risk factors separately for each of subgroups. Because the reference group is representative of the target group, these derived goal levels of risk factors for each of the subgroups based on the data from the reference group can be adopted as the respective goal levels for the respective subgroups of the target group. Prevention settings to achieve the goal levels of all risk factors for each participant in the target group can then be designed individually based on his/her measured risk profile from the screening/baseline exam, respective subgroup goal levels, and prevention program. Individuals in each subgroup of the reference group will be classified as positive (if their “predicted incident risk from the prediction model” ≥ the given cutoff probability p*) or not-positive (other-wise). For each subgroup and a continuous risk factor, we propose to use a regression model to derive the goal level for the risk factor. In the regression model, the risk factor is the dependent variable, and the other risk factors in the prediction model and a classified variable (=1 if an individual is positive; =0, otherwise) are independent variables. Least-squares means (LSM) and 95% confidence interval (CI) of the risk factor for those positives and not-positives in the subgroup then can be estimated from the regression. The LSM represents the mean of the risk factor after adjusting for the other risk factors since they may be correlated. We propose to use the upper bound of the 95% CI of the LSM of the risk factor from those not-positives in the sub-group as the goal level of the risk factor for the subgroup (the lower bound will be used if the risk factor is negatively associated with the disease in the prediction model). For a dichotomous risk factor, a similar procedure using a logistic regression model will be applied. It is obvious that if the participants in each subgroup of the target group approach the goal levels of the risk factors for the subgroup through the prevention program, that is, their levels of risk factors will not differ significantly from those of not-positives, consequently their expected disease risks will also decrease and approach the risks of those who are not positive.

For example, the regression model for deriving the upper bound of the 95% CI of the LSM of FPG from those not-positives in a subgroup (the goal level of risk factor FPG for the subgroup) is as follows.

(6) $$\begin{array}{l} \text{FPG}=b_{0}+b_{1}\times I(\text{individual is positive})+b_{2}\times \text{Age}+b_{3}\times \text{WAIST}\\ \;+\;b_{4}\times I(\text{HTN medications})+b_{5}\times \text{SBP}+b_{6}\times \text{DBP}\\ \;+b_{7}\times \text{HbAlc}+b_{8}\times \mathrm{Log}(\text{UACR})+b_{9}\times \mathrm{Log}(\text{TG})+\varepsilon \end{array}$$

where *ε* denotes the error term and *I*(.) is the indicator function.

### 2.3. Assessments

Let APPDM_positive,*i*_ and APPDM_not-positive,*i*_ denote the estimated average predicted probabilities of developing DM (PPDM) in four years from those positives and not-positives in the ith subgroup of the reference group, respectively; and *m*_*i*_ and *k*_*i*_ denotes the number of positives (intervention participants) and not-positives, respectively, in the *i*th subgroup of the target group. Then, two APPDMs for a subgroup can be used to pre-assess expected intervention effects for the subgroup. In addition, the weighted average

(7) $$\sum_{i}m_{i}{\text{APPDM}}_{\text{positive,}i}/\sum_{i}m_{i}-\sum_{i}k_{i}{\text{APPDM}}_{\text{not-positive,}i}/\sum_{i}k_{i}$$

will give the pre-assessed expected intervention effect for all intervention participants. Furthermore, the difference between PPDM based on the risk factor measurements at the screening/baseline exam for prevention and at the exam at the end of the intervention period from each intervention participant can be used as a score to estimate the true prevention effect.

## 3. Results

The characteristics for baseline participants of the SHS have been reported previously [5]. Based on those Example, Available result and Already collected data defined in the Methods section, and applying Equation (3a), (4) and the methods explained in the Methods section, Table 1 gives the derived p* for different assumed CBR based on the data from the reference group and the SHS DM prediction model. For instance, when CBR = 0.406 (=CIDM/(1-CIDM)), the corresponding cutoff probability p* = 0.2945. If this p* will be used in identification, by using the measured risk factors at the screening exam, those AI in the target group whose predicted probability (from Equation (1)) ≥ p* (=0.2945) would be classified as “at high risk of developing DM” or “positive” and be selected for intervention.

Based on the data from the reference group and Equation (5), Table 1 showed that the previous p* = 0.2945 is also the affordable cutoff probability if the budget is limited to have only 38% of AI in the target group receive the intervention.

According to the methods explained in the Methods section, we divide all individuals in the reference group into four subgroups (FPG ≤ 106 mg/dL and HbAlc ≤ 5.3%, FPG ≤ 106 mg/dL and HbAlc 5.4% - 6.4%, FPG 107 – 125 mg/dL and HbA1c ≤ 5.3%, FPG 107 – 125 mg/dL and HbA1c 5.4% - 6.4%) based on the 50th percentiles of FPG (106 mg/dl) and HbA1c (5.3%). Table 2 gives the information and the simultaneous goal levels (the bolded upper bound of 95% CI from those not-positive) of all risk factors in the SHS DM prediction model, for CBR = 0.406 (=CIDM/(1-CIDM)) (or equivalently, p* = 0.2945) and the four subgroups. To use Table 2 in the DM prevention, say, at the screening exam, those AI in the target group, who would be identified as positive (“predicted probability” ≥ p* = 0.2945) and belonging to the last subgroup (FPG in 107 – 125 mg/dl and HbA1c > 5.3%) in Table 2, should reduce/keep their FPG, HbA1c, UACR, TG and WAIST levels below the goal levels of 112 mg/dl, 5.6%, 6 mg/g, 125 mg/dl and 113 cm, respectively; and SBP/DBP below 129/77 mmHg if not on HTN medication treatments to prevent DM. The reductions in TG and SBP/DBP are also implied the participants in this subgroup should not have either elevated TG or elevated blood pressures, or should reduce their rates of elevated TG and elevated blood pressures below the goal rates of 13.22% and 51.96% (Table 2), respectively, to prevent incident DM.

**Figure 1.**
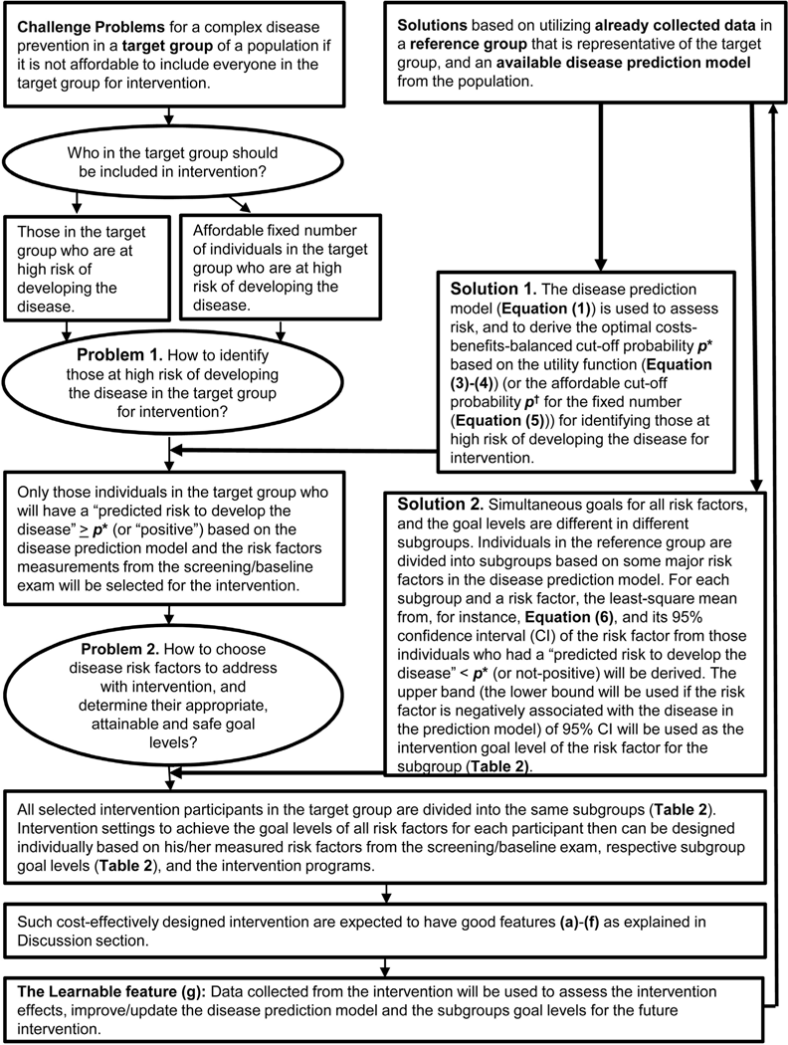
The challenges and our proposed solutions for designing a complex disease prevention.

Figure 1 provides a summary diagram of the proposed design and strategy.

## 4. Discussion

Implementing a disease prevention intervention for all individuals in a target group is usually not economically affordable, or may result in unnecessary intervention for large percent of individuals with low risk [13]. For examples, among those AIs who participated the SHS, the proportions of those potential participants for DM interventions considered in the literature such as pre-DM or obese [2] [3] [17] [18] [19] were about 14% or 51%, respectively. But, only about 27% of the pre-DM and 28% of the obese AI participants later developed DM in an average of 4 years. We proposed to use an available disease prediction model from the same population to assess risk for taking account of the combined effects of risk factors, and to use the optimal costs-benefits-balanced cutoff probability for selecting intervention participants to minimize false-positive and maximize true-positive assignment of intervention participants to balance the costs and benefits. Compared to an intervention for all AI in the target group (about only 29% of them might develop DM later if without intervention based on the data from the reference group), the intervention for those positive AI identified by the cutoff probability p* = 0.2945 (about 45% of them later might develop DM if without intervention) is clearly more efficient. In addition, we also proposed the cutoff probability (Equation (5)) for identifying those who are “positive” in case that budget allows only an affordable number of individuals in the target group for the intervention.

Recent clinical trials demonstrated that lifestyle/pharmaceutical interventions may prevent development of DM [17] [20] [21]. However, the question of how a DM prevention should be monitored is not clear [11]. Compared with the usual way of setting uniform goal levels for one/two risk factors for all participants in an intervention, we adopted the ways from our previous paper [16] to conduct simultaneous intervention for all risk factors in the disease prediction model and to set goal levels for all risk factors and vary them for different subgroups. Our approach has the following features as we explained also in the previous paper.

a) Addressed complex associations of a disease with its combined and correlated major risk factors, and used all available valuable results and costly collected data in the design.
b) It is reasonable to expect that individuals in the same subgroup have approximately similar health conditions. The proposed goal levels based on the levels of risk factors from those not-positives in the same subgroup accommodate subgroup differences and the combined and correlated effects of the DM risk factors. Therefore, these proposed goal levels might be more appropriate, attainable and safe compared to those usual ways of setting uniform goal levels for all participants in an intervention. Moreover, in an intervention, for a participant in a subgroup, if his/her levels of some risk factors are already satisfying the respective goal levels, no interventions for these risk factors will be conducted, and thus is cost-saving.
c) The derived information and goal levels (Table 2) can be used for the awareness of a disease, risk factors of the disease, and intervention effects for health providers and participants. For example, in the last subgroup in Table 2, the LSM of FPG, HbA1c, UACR and WAIST, the hypertriglyceridemia and elevated-blood-pressure rates between positives and not-positives were significantly different. Thus these risk factors are the reasons why some individuals in this subgroup were positive while the others were not, and thus should get more attention in intervention. Moreover, the estimated average predicted probabilities of developing DM (APPDM) in four years for positives and not-positives in different subgroups based on the data from the reference group can also be used to show potential intervention benefits. For example, for positives (those intervention participants) in the target group who belong to the last subgroup in Table 2, their APPDM might be 45.6% if without intervention. However, if they approach all their goal levels through the intervention, their APPDM might be reduced to 24.9% (the level of those not-positives).
d) Table 2 shows a suggestion for a gradual intervention. For example, the 3rd and 4th subgroups were defined by the same FPG range but different HbA1c ranges, and the goal levels for HbA1c were gradually relaxed from <5.0% to <5.6%. Therefore, in intervention, an individual belonged to the 4^th^ subgroup would be instructed to reduce/keep his/her level of HbA1cto <5.6%, while the 3rd subgroup <5.0%. Of course, participants in the 4^th^ subgroup would not be discouraged to reduce their level of HbA1c to <5.0% (the goal for the 3^rd^ subgroup), but they could do this gradually (first <5.6% then <5.0%) and thus safer and more attainable. This feature may reduce frustrations of participants who have more serious health conditions but be stressed to quickly reduce their risk factor levels to those usual uniformed goal levels for everyone in an intervention. This feature may be necessary considering a chronic disease is a chronic and cumulative outcome of combined risk factors, and therefore the return to normal levels of the risk factors should be also a gradual process that occurs over time.
e) Table 2 shows an adaptive strategy for the intervention. For example, if an individual belongs to the last subgroup (FPG in 107 – 125 mg/dl and HbA1c > 5.3%) at the beginning of the intervention and his/her HbA1c is later reduced to ≤5.3% while FPG remained unchanged during the intervention, and the improved HbA1c remains stable in perhaps two consecutive visits, then his/her goal levels and intervention settings could be adaptively changed to those in the subgroup with FPG in 107 – 125 mg/dl and HbAlc ≤ 5.3%.
f) Easy prediction and assessments for the intervention as explained in Methods section.
g) Learnable. Data collected from the intervention might be added to the already collected data, and the expanded data then might be used to improve/update the disease prediction model and the subgroup goal levels for the future intervention.

We proposed and demonstrated how to utilize and translate the available research results from SHS in the cost-effective design of a DM prevention program for the target group, and assessed/predicted the effectiveness of our proposed strategy. The strategy and methods shown in the illustrative example for DM prevention can be analogously adopted and applied for other disease preventions. To our knowledge, the proposed cost-effective design strategy is new representing a novel frame work for the utilization and translation of large collected data to inform practice. However, such design strategies need to be tested and validated in real disease prevention studies. The proposed strategy depends on a disease prediction model and risk factors data from the same (or similar) population of the target group. If the needed information is not available from the same population, one may use available information from another population that closely resembles the population under study. The cutoff probability p* from Equation (4) depends on assumed/estimated CBR. The estimation of CBR depends on intervention programs and the definitions of costs and benefits [2] [3]. Only four subgroups were demonstrated in Table 2 due to the limited sample size. We may expect the learnable feature (**g**) of our strategy will allow us to define more subgroups and thus set more appropriately individualized goal levels in the future.

A limitation specific, not to the approach, but to the disease diabetes is that the two risk factors that are more cost effective are not on the causal path to the development of type 2 diabetes. Elevated triglycerides and blood pressure levels are a result of the insulin resistance that is the determinant that results in elevated glucose levels and eventual pancreatic fatigue. It is not feasible to measure insulin resistance in a clinical setting, however. Thus correcting the elevated triglycerides and blood pressure may not improve insulin resistance. This limitation is specific to diabetes, however, whereas in most other chronic diseases, such as cardiovascular disease, the measurable risk factors are in the causative pathway (e.g. elevated LDL C). Thus, the strategy presented here may be even more cost effective in those cases.

## 5. Conclusion

The proposed strategy considers the complex associations of a disease with its combined and correlated risk factors and individual differences; provides ways to cost-effectively identify individuals for intervention, and to simultaneously set gradual, attainable and safe goal levels for all risk factors in different subgroups; and forms an adaptive intervention frame. The proposed design strategy represents a way to utilize or translate available valuable results and costly collected data from large cohort studies for clinical disease prevention practice, and can be applied to group/community disease prevention interventions.

## Figures and Tables

**Table 1.** Optimal costs-benefits-balanced cutoff probability p* for different assumed costs-to-benefits ratios (CBR).

	CBR					
	0.406 (=CIDM/(1-CIDM))	0.2	0.4	0.6	0.8	1

p*	0.2945	0.148	0.295	0.302	0.404	0.468
%^a^	38.30%	83.70%	38.30%	36.80%	16.50%	9.30%

a The respective expected percentage of American Indians in the target group who will be identified as “at high risk of developing DM” or “positive” by using the p* in the screening exam, and hence will be included for DM intervention. CIDM, estimated cumulative incidence rate of DM in 4 years in the target group (CIDM = 0.2888, based on the data from the reference group, and hence CIDM/(1-CIDM) = 0.4060); DM is defined as FPG ≥ 126 mg/dl or HbAlc ≥ 6.5%.

**Table 2.** For optimal costs-benefits-balanced cutoff probability p* = 0.2945, suggested intervention goal levels (bolded upper bound of 95% CI) for DM risk factors.

FPG	HbA1c		Not-Positive			

(mg/ dl)	(%)	Risk Factor	LSM	95% CI	P^a^

≤106	≤5.3	FPG (mg/dl)	97	96	**98**	0.0595
		HbAlc (%)	4.9	4.8	**4.9**	**0.0164**
**Not-Positive**	**Positive**	UACR (mg/g)	6	5	**7**	**0.0001**
n = 257	n = 21	TG (mg/dl)	113	107	**120**	**0.0019**
APPDM = 0.164 APPDM = 0.356		TG ≥ 150 mg/dl	21.7%	16.81%	**27.48%**	**0.0002**^c^
APPDM-All	= 0.178	SBP/DBP ≥ 130/85 mmHg or on medication for HTN	59.7%	53.16%	**65.98%**	**0.0176**^c^
		DBP^b^ (mmHg)	77	76	**78**	**0.0037**
		SBP^b^ (mmHg)	123	121	**124**	**0.0033**
		WAIST (cm)	112	111	**113**	**<0.0001**

≤106	5.4 – 6.4	FPG (mg/dl)	97	96	**98**	**0.0005**
		HbAlc (%)	5.6	5.6	**5.7**	**<0.0001**
**Not-Positive**	**Positive**	UACR (mg/g)	7	5	**10**	0.0607
n = 79	n = 69	TG (mg/dl)	117	105	**129**	0.1262
APPDM = 0.210 APPDM = 0.405		TG ≥ 150 mg/dl	13.3%	6.57%	**25.02%**	**0.0076**
APPDM-All = 0.301		SBP/DBP ≥ 130/85 mmHg or on medication for HTN	46.9%	30.94%	**63.44%**	**0.0054**
		DBP (mmHg)	74	72	**76**	**0.0314**
		SBP (mmHg)	122	119	**125**	0.0717
		WAIST (cm)	112	110	**115**	**0.0003**

107 – 125	<5.3	FPG (mg/dl)	112	111	**113**	**<0.0001**
		HbA1c (%)	4.9	4.9	**5.0**	0.3036
**Not-Positive**	**Positive**	UACR (mg/g)	6	5	**8**	**0.0255**
n = 114	n = 63	TG (mg/dl)	115	106	**125**	**0.0002**
APPDM = 0.218 APPDM = 0.360		TG ≥ 150 mg/dl	7.7%	3.79%	**14.98%**	**<0.0001**
APPDM-All = 0.268		SBP/DBP ≥ 130/85 mmHg or on medication for HTN	35.8%	25.22%	**48.04%**	**<0.0001**
		DBP (mmHg)	75	74	**77**	0.0549
		SBP (mmHg)	120	118	**123**	**0.0011**
		WAIST (cm)	111	110	**113**	**<0.0001**

107 – 125	5.4 – 6.4	FPG (mg/dl)	111	109	**112**	**<0.0001**
		HbA1c (%)	5.6	5.5	**5.6**	**<0.0001**
**Not-Positive**	**Positive**	UACR (mg/g)	3	2	**6**	**0.0019**
n = 39	n = 151	TG (mg/dl)	108	93	**125**	0.0940
APPDM = 0.249 APPDM = 0.456		TG ≥ 150 mg/dl	4.2%	1.22%	**13.22%**	**0.0002**
APPDM-All = 0.413		SBP/DBP ≥ 130/85 mmHg or on medication for HTN	32.2%	17.27%	**51.96%**	**0.0017**
		DBP (mmHg)	75	72	**77**	0.6384
		SBP (mmHg)	125	121	**129**	0.8488
		WAIST (cm)	111	108	**113**	**<0.0001**

a p-value from testing the difference of least-square means between positive and not-positive AI in a sub-group.

b The results for DBP and SBP are based on data from those without hypertension medications treatments.

c p-value from testing the difference of least-square rates of the metabolic syndrome trait between positive and not-positive AI in a subgroup. AI, American Indians; CI, confidence interval; DBP, diastolic blood pressure; n, the sample size; APPDM, estimated average predicted probability of developing DM in four years; FPG, fasting plasma glucose; HbAlc, hemoglobin A1c; HTN, hypertension; LSM, least-square mean; SBP, systolic blood pressure; TG, triglycerides; UACR, urinary albumin and creatinine ratio; WAIST, waist circumference.
